# Does Elective Admission vs. Emergency Department Presentation Affect Surgical Outcomes in Metastatic Spine Surgery?

**DOI:** 10.3390/diagnostics14101058

**Published:** 2024-05-20

**Authors:** Tyler Zeoli, Hani Chanbour, Ranbir Ahluwalia, Amir M. Abtahi, Byron F. Stephens, Scott L. Zuckerman

**Affiliations:** 1 Department of Neurological Surgery, Vanderbilt University Medical Center, Nashville, TN 37232, USA; tyler.zeoli@vumc.org (T.Z.);; 2 Department of Orthopedic Surgery, Vanderbilt University Medical Center, Nashville, TN 37232, USA

**Keywords:** spinal tumors, presentation, emergency department, clinic, outcomes, overall survival

## Abstract

Introduction: Approximately 20% of patients with metastatic spine disease develop symptomatic spinal cord compression, and these patients can present urgently to the emergency department (ED) or, in a more organized fashion, to a clinic. In a cohort of patients undergoing metastatic spine surgery, we sought to (1) determine the rate of ED presentation, (2) identify preoperative and perioperative risk factors associated with ED presentation, and (3) evaluate whether ED vs. clinic presentation impacts long-term outcomes. Methods: A single-institution, multi-surgeon, retrospective cohort study was undertaken of patients undergoing metastatic spinal tumor surgery between 02/2010 and 01/2021. The primary exposure variable was presentation setting, dichotomized to the ED vs. clinic. The primary outcomes were postoperative functional status, measured with the Karnofsky Performance Scale (KPS) and McCormick Scale (MMS), local recurrence (LR), and overall survival (OS). Secondary outcomes included complications and readmissions. Results: A total of 311 patients underwent metastatic spine surgery (51.7% ED vs. 48.3% clinic). Those presenting to the ED had higher rates of smoking (21.7% vs. 16.0%, *p* = 0.02), were more likely to have 2+ comorbidities (47.2% vs. 32.7%, *p* = 0.011), and were more likely to have public insurance (43.5% vs. 32.0%, *p* = 0.043). Preoperative KPS was lower in ED patients (*p* < 0.001), while the Bilsky score was higher (*p* = 0.049). ED patients had higher rates of oligometastatic disease (*p* = 0.049), higher total decompressed levels (*p* = 0.041), and higher rates of costotransversectomy (*p* = 0.031) compared to clinic patients. Length of stay was significantly longer for ED patients (7.7 ± 6.1 vs. 6.1 ± 5.8 days, *p* = 0.020), and they were less likely to be discharged home (52.2% vs. 69.3%, *p* = 0.025). ED presentation was significantly associated with shorter overall survival (HR =1.53 95% CI = 1.13–2.08, *p* = 0.006). Conclusions: Of patients undergoing metastatic spine disease, approximately half presented through the ED vs. clinic. ED patients had higher rates of smoking, public insurance, and higher Bilsky score. ED patients also underwent more extensive surgery, had longer LOS, were less likely discharged home, and most importantly, had a shorter overall survival. These results suggest that initial presentation for patients undergoing surgery for metastatic spine disease significantly impacts outcomes, and signs/symptoms of metastatic spine disease should be recognized as soon as possible to prevent ED presentation.

## 1. Introduction

The incidence of metastatic spine disease continues to increase despite improved cancer treatment options [1,2], and the spinal column remains the most common site of osseous metastasis [1,3]. With roughly 33–70% [1,3,4] of solid cancer patients developing spinal involvement, nearly 20% of these patients will develop symptomatic spinal cord compression [1,3,4,5]. Surgical intervention for these symptomatic lesions has shown to be effective in improving neurological outcomes, pain, and even quality of life [1,5,6].

Patients with metastatic spine disease, with or without cord compression, can present either in an organized fashion to a clinic or in a more urgent manner to the emergency department (ED). When an acute neurologic deficit occurs, ED evaluation is necessary. However, neurologically intact patients with mechanical pain can also present to the ED in a less urgent manner. In a retrospective analysis of over 300 patients undergoing metastatic spine surgery, Zanaty et al. 2022 [7] showed the majority presented emergently through the ED, with that same cohort having a longer length of stay (LOS) and lower average survival compared to those presenting to the clinic [7]. Moreover, some current research suggests that involvement of a tumor board in patients with spinal metastasis can decrease the rate of ED presentation, the need for emergent surgery, and improve outcomes [2]. Thus, it is imperative to assess risk factors that predispose patients to present emergently through the ED and also compare overall outcomes after surgical intervention.

Given the different presentation settings of patients undergoing surgery for metastatic spine disease, gaining a better understanding of how and why these patients present may improve care. Therefore, in a cohort of patients undergoing metastatic spine surgery, we sought to (1) determine the rate of ED presentation, (2) identify preoperative and perioperative risk factors associated with ED presentation, and (3) evaluate whether ED vs. clinic presentation impacts long-term outcomes.

## 2. Methods

### 2.1. Study Design

A single-institution, multi-surgeon, retrospective case-control study was undertaken for patients undergoing metastatic spinal tumor surgery between February 2010 and January 2021. Institutional review board (IRB) approval was obtained for this study (IRB#211900).

### 2.2. Patient Population

Registry data were selected for patients who underwent surgery for spinal metastasis between 2010 and 2021. Inclusion criteria were adult patients (18 years old or above) with metastatic extradural spinal tumors who underwent spinal surgery for tumor resection or stabilization. Exclusion criteria consisted of pediatric patients (less than 18 years old), intradural tumors, and primary tumors. The date of the last follow-up was extended to the date of death or the date of the last clinical follow-up.

### 2.3. Exposure Variable

The primary exposure variable was presentation setting, dichotomized as either the clinic or the ED. The clinic setting was a priori, defined as a surgeon’s office, directly referred by an oncologist, radiation oncologist, or primary care physician. The ED was defined as any urgent care or emergency room setting, or inter-hospital transfer. Additional exposure variables were divided into preoperative and perioperative variables. Preoperative variables included demographics of age, sex, and body mass index (BMI). Other factors included associated comorbidities, smoking status, insurance type, and primary organ disease. Perioperative variables included pain status (mechanical, biologic, neurologic), motor/sensory deficits, tumor size and location, preoperative Karnofsky Performance Scale (KPS), Bilsky Score, and preoperative chemotherapy or radiotherapy. Other variables included type of surgery, total instrumented levels, total decompressed levels, estimated blood loss, intraoperative monitoring changes, operative time, length of stay (LOS), discharge disposition, and complications.

### 2.4. Outcome Variable

The primary outcomes of interest included postoperative outcomes of functional status, measured with the Karnofsky Performance Scale (KPS) and McCormick Scale (MMS), local recurrence (LR), and overall survival (OS). Secondary outcomes included complications and readmissions.

### 2.5. Surgical Treatment

All patients underwent surgery in accordance with the strategy of separation surgery, consisting of spinal cord decompression and long-segment posterior stabilization and fusion [8,9,10,11]. Patients were most often taken for a posterior approach, potentially involving a transpedicular approach or costotransversectomy to achieve adequate spinal cord decompression. The goal of adequate spinal cord decompression was to achieve a separation between the tumor and the spinal cord, in addition to reconstituting the thecal sac, to achieve a safe distance from the tumor to the spinal cord for adequate radiation dosing. Ultrasound was used often to confirm adequate spinal cord decompression. Anterior column reconstruction was sometimes performed depending on the extent of kyphosis, the presence of a lytic lesion, and surgeon preference.

### 2.6. Statistical Analysis

Descriptive statistics were reported to compare preoperative and postoperative variables. The mean and SD were reported for continuous variables and frequency for categorical variables. Normal distribution and variance for continuous variables were assessed with the Shapiro–Wilk test and F test, respectively. Parametric data with equal variance were analyzed with a two-tailed t-test, while nonparametric data were compared with the Wilcoxon signed rank or Mann–Whitney test. The χ^2^ or Fisher exact test was used for nominal data. A Kaplan–Meier plot, as well as univariable/multivariable logistic/cox/linear regressions, were performed, controlling for age, comorbidities, preop KPS, the primary organ of origin, and the presence of other organ metastases. An α value < 0.05 was considered statistically significant. All analyses were performed using R, version 4.1.3 (The R Foundation, Vienna, Austria).

## 3. Results

### 3.1. Patient Demographics

**Figure 1:**
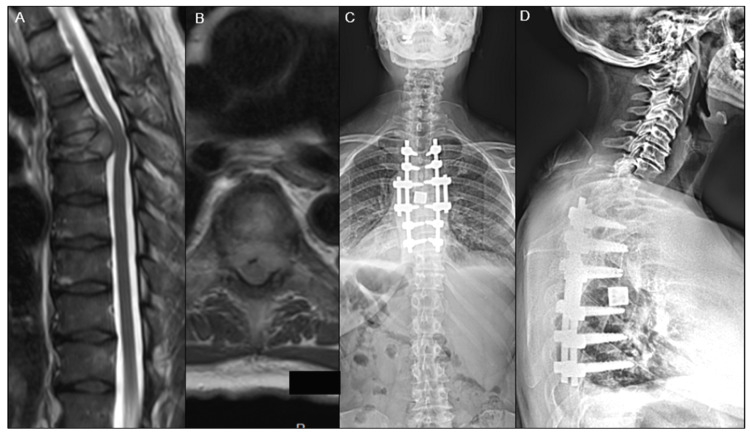
A 53-year-old female presented to the clinic with a several-week history of back pain and difficulty ambulating due to poor balance. On preoperative T2-weighted MRI (**A**,**B**), the patient was found to have T6 Bilsky grade 3 epidural spinal cord compression due to metastatic breast cancer. The patient underwent separation surgery with T3-T9 posterolateral fusion, T5-T7 laminectomies, T5-T6 and T6-T7 osteotomies, inferior facetectomies at T3-T9, costotransversectomy on the left side at T6 with removal of 4 cm of the left T6 rib, and bilateral transpedicular decompression at T6 with T4-T7 anterior fusion with a static cage filled with allograft and demineralized bone matrix, as seen on postero-anterior (**C**) and lateral X-ray (**D**).

**Figure 2:**
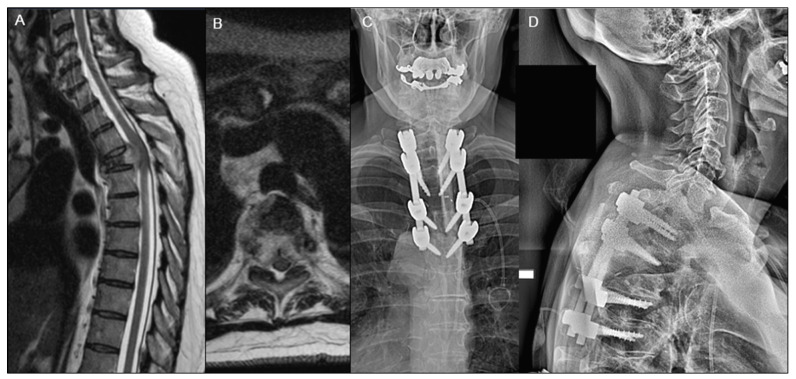
A 61-year-old female patient presented to the emergency department with bilateral leg weakness and right shoulder pain and was found to have Bilsky 3 compression at T4 as seen on preoperative T2-weighted MRI (**A**,**B**). The patient underwent separation surgery with thoracic laminectomy, partial of T3, total of T4, with transpedicular corpectomy bilaterally of the T4 vertebral body with greater than 50% removal of the T4 vertebral body and decompression of the spinal cord as seen on her postero-anterior and lateral X-rays (**C**,**D**). Despite initial improvement, the patient died a year later from progression of her renal cell carcinoma.

A total of 311 patients underwent surgery for spine metastasis (51.7% ED vs. 48.3% clinic) with a mean follow-up time of 516.6 ± 634.1 days. Overall, 186 (59.8%) were males, and 170 (54.7%) had other organ metastases. The mean age at surgery for patients presenting to the ED was 62.1 ± 11.8 years compared to clinic patients 59.0 ± 12.1 years (*p* = 0.023). There was no significant difference found in baseline demographics, including sex (*p* = 0.133), race (*p* = 0.684), and BMI (*p* = 0.278) (Table 1). There was no difference in preoperative neurological deficits from a sensory or motor perspective. Representative cases of a patient admitted from the clinic and a patient admitted from the ED are described in Figure 1 and Figure 2.

### 3.2. Preoperative Factors Associated with ED Presentation

Those presenting to the ED had higher rates of smoking (21.7% vs. 16.0%, *p* = 0.022), were more likely to have two or more comorbidities (47.2% vs. 32.7%, *p* = 0.011), and were more likely to have public insurance (43.5% vs. 32.0%, *p* = 0.043) compared to clinic patients. Moreover, patients presenting to the ED had higher rates of biologic pain (60.2% vs. 38%, *p* < 0.001) compared to higher reported neurological pain for clinic patients (47.3% vs. 36.0%, *p* = 0.050). Preoperative KPS was lower in patients presenting to the ED (62.7 ± 17.75 vs. 70.0 ± 14.6, *p* < 0.001), while the Bilsky score was higher (*p* = 0.049). In addition, ED patients had higher rates of oligometastatic disease (*p* = 0.049). There were no differences in reported mechanical pain (*p* = 0.909), sensory or motor deficits (*p* = 0.108; *p* = 0.139), tumor size (*p* = 0.348), preoperative embolization rates (*p* = 0.088), or preoperative chemotherapy or radiotherapy (*p* = 0.066; *p* = 0.074) (Table 2 and Table 3).

### 3.3. Perioperative Factors Associated with ED Presentation

Patients presenting to the ED had higher total decompressed levels (*p* = 0.041) and higher rates of costotransversectomy (*p* = 0.031) compared to clinic patients. There were no statistical differences found in the total instrumented levels (*p* = 0.227), operative time (*p* = 0.367), estimated blood loss (*p* = 0.122), intraoperative monitoring changes (*p* = 0.999), or rates of transpedicular decompression (*p* = 0.497) (Table 3). Length of stay was significantly longer for ED patients (7.7 ± 6.1 days vs. 6.1 ± 5.8 days, *p* = 0.020), and they were less likely to be discharged home (52.2% vs. 69.3%, *p* = 0.025) (Table 4).

### 3.4. Long-Term Outcomes

**Figure 3:**
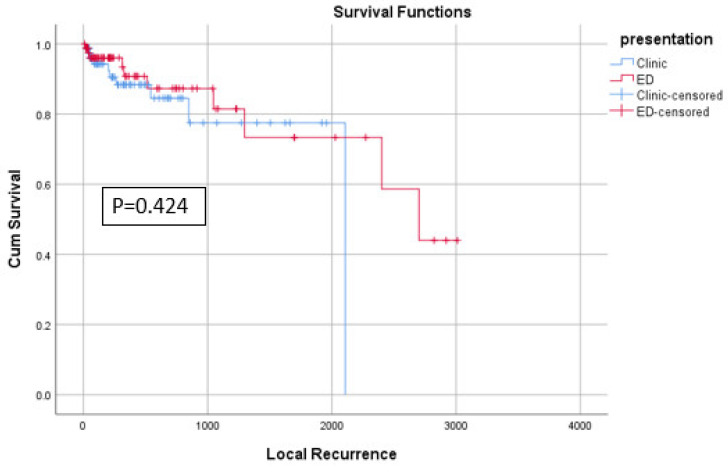
KM plot of local recurrence.

**Figure 4:**
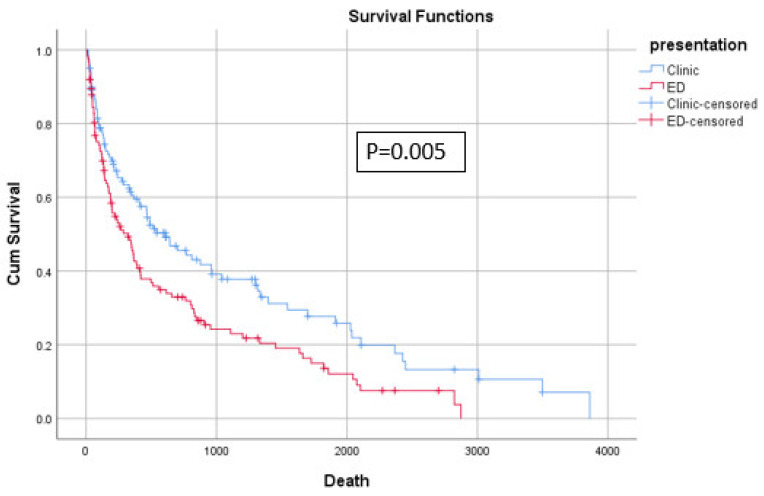
KM plot of overall survival.

For our primary outcomes, no significant difference was found regarding local recurrence (*p* = 0.716), time to local recurrence (log-rank test = 0.424) (Figure 3), KPS at last-follow-up (*p* = 0.127), and MMS at last follow-up (*p* = 0.428) (Table 5). However, patients presenting to the ED had a shorter time to death (458.2 ± 604.3 vs. 481.6 ± 749.9 days, *p* = 0.005) (Figure 4). On Multivariable Cox regression controlling for other covariates such as age, comorbidities, preop KPS, primary organ of origin, and the presence of other organ metastasis, ED presentation was significantly associated with shorter overall survival (HR = 1.53, 95%CI = 1.13–2.08, *p* = 0.006) (Table 6).

### 3.5. Complications

For our secondary outcomes of complications and readmissions, we found no difference between groups in complications (*p* > 0.999), readmission (*p* = 0.443), or reoperation (*p* = 0.588). Postoperative complications are summarized in Table 4. The most common complications were pneumonia (5.5%) and wound breakdown (5.5%), which were not different across groups. There was no difference in postoperative chemotherapy or radiotherapy (*p* = 0.244; *p* = 0.357) or rates of mechanical complications (*p* = 0.322). New postoperative neurological deficit was not significantly different in ED and clinic patients (*p* = 0.113).

## 4. Discussion

The current study assessed patients undergoing surgical intervention for metastatic spine disease and determined risk factors and differences between patients who presented to the ED vs. clinic. Patients presenting to the ED were older, had more comorbidities, and more often had public insurance. ED patients also had a lower preoperative KPS score and higher Bilsky score, though the rate of preoperative neurologic deficits was similar. Operatively, ED patients underwent larger decompressions and, more often, costotransversectomies with a longer LOS. Despite similar rates of postoperative chemotherapy and RT and controlling for several confounding variables, ED patients had a significantly decreased OS. These results shed light on the less healthy and overall higher-risk patient population that ultimately presents to the ED with lower overall survival compared to clinic patients.

Patients presenting through the ED had more comorbidities, higher rates of public insurance, higher Bilsky scores, and higher rates of biologic pain. The current literature has clearly established insurance type as a mediator of prehospital management and overall outcomes in oncology patients [12,13]. In a study focusing on the impact of public insurance on tumor burden, Price et al. [12] found that those with public insurance had higher rates of metastatic spinal cord compression and paralysis, were less likely to receive surgery, and had longer hospital stays. While our study did not find differences in the degree of compression via the Bilsky score, we found those presenting to the ED were more likely to have public insurance, which points toward socioeconomic factors that may predispose these patients to less optimal resources and pre-surgical follow support, thus leading to worse outcomes. Biologic pain is often associated with increased inflammation, and knowing that tumor invasion often leads to inflammatory cascades within surrounding tissue [14,15], we suspect that this finding suggests a larger tumor burden with a more rapidly progressed disease for those presenting to the ED. This complex interplay between lower socioeconomic status, more advanced disease, and presenting to the ED highlights a potential area of research. A broader discussion of addressing disparities in care may serve as fruitful to improve the care of metastatic spine patients.

Regarding perioperative differences between ED and clinic patients, we found a more extensive decompression required in the ED patient population. The requirement of a more robust surgical intervention may be due in part to a higher tumor burden creating destruction to surrounding soft tissue and bone through local invasion, thus necessitating a more extensive decompression of neural elements. The most commonly reported complication of wound breakdown was in line with what our data suggests [10].

Despite no significant difference in postoperative KPS and MMS, our data suggests a longer hospital stay for surgical patients presenting through the ED. This discrepancy is further highlighted by the fact that the ED patient cohort was less likely to be discharged and, thus, more likely to be sent to a facility. Given there were no differences in neurological deficits or other surgical complications, this suggests less home support for patients presenting to the ED, which makes sense given the unexpected need for postoperative care at home. Of note, KPS was improved postoperatively for both cohorts, which validates the reasons for surgery. However, it declined at the time of the last follow-up, which presents the natural progression of patients with metastatic spinal tumors.

Lastly, and perhaps our most notable findings, patients undergoing spinal metastatic surgery who presented to the ED had lower odds of survival compared to those presenting for elective surgery through a clinic. Despite controlling for several confounding variables, including postoperative chemotherapy and radiation, ED patients had lower odds of survival. Reasons for this are multifactorial but may relate to the lower socioeconomic status of these patients or a more rapidly progressing disease. Thus, it is imperative to understand the perioperative risk factors that lead to ED presentations to improve outcomes and survival among this patient population. Once patients’ symptoms reach the point of requiring ED admission, the battle may have already been lost. Further research should focus on ways to detect metastatic spine disease during surveillance of patients with cancer and educating patients on what to look for in those without cancer but with strong family histories of such.

There are several limitations to this study, which may limit the generalizability of the results. First, we did not identify clinic patients who were sent directly to the ED, which is a population of patients that would be interesting to separately identify and analyze and could have confounded results. Second, there are other factors that could influence patient outcomes, including time to surgery, type of instrumentation, length of surgery, drain management, and type of radiation. Furthermore, a sub-analysis of the primary organ of origin could not be performed due to the small sample size. Third, there was limited data regarding patient presentation to other hospitals postoperatively, which could alter our findings on wound complications, readmission, and reoperation rates. Fourth, the overall prognosis postoperatively depends widely on targeted treatment modalities specific to the genetic profiles of the patient’s primary tumors. The molecular basis of the patient’s primary tumor was not included in our study and thus limits our postoperative analysis. We aim to include molecular data in future studies, as this is an important and growing area of spinal oncology. Fifth, the study was performed at a single academic institution within the United States, and thus, it is possible these findings may not reflect the general population. This ultimately limits our generalizability to differing healthcare systems abroad, such as publicly managed national health systems. A larger multicenter and multinational study should be designed to uncover additional factors associated with ED vs. clinic presentation for metastatic spine surgery.

## 5. Conclusions

In patients undergoing spine surgery for metastatic disease, half of the patients were admitted urgently through the ED rather than the clinic, with those in the former group exhibiting higher rates of smoking, reliance on public insurance, and more severe Bilsky scores. Notably, ED patients underwent more extensive surgical procedures and had worse postoperative outcomes. These findings underscore the significant influence of the initial presentation on surgical outcomes, highlighting the crucial need for early recognition of symptoms to prevent ED admissions.

## Figures and Tables

**Table 1:** Demographic variables.

	Total *N* = 311	ED *N* = 161	Clinic *N* = 150	*p*-Value
Age mean ± SD	60.6 ± 12.0	62.1 ± 11.8	59.0 ± 12.1	0.023
BMI mean ± SD	27.1 ± 7.0	26.7 ± 7.0	27.6 ± 6.9	0.278
Gender, Male *n* (%)	186 (59.8%)	103 (64.0%)	83 (44.3%)	0.133
Race, white *n* (%)	271 (87.1%)	140 (87.0%)	131 (87.3%)	0.684
Insurance, *n* (%)				0.043
Private	112 (36.0%)	48 (29.8%)	64 (42.7%)	
Public	118 (37.9%)	70 (43.5%)	48 (32.0%)	
Uninsured	81 (26.0%)	43 (26.7%)	38 (25.3%)	
Hypertension, *n* (%)	173 (55.6%)	94 (58.4%)	79 (52.7%)	0.361
HLD, *n* (%)	65 (20.9%)	37 (23.0%)	28 (18.7%)	0.403
Diabetes, *n* (%)	72 (23.2%)	40 (24.8%)	32 (21.3%)	0.503
Osteoporosis, *n* (%)	9 (2.9%)	4 (2.5%)	5 (3.3%)	0.743
Cardiac, *n* (%)	58 (18.6%)	33 (20.5%)	25 (16.7%)	0.467
Psychiatric, *n* (%)	56 (18.0%)	28 (17.4%)	28 (18.7%)	0.770
Comorbidities 2+, *n* (%)	125 (40.2%)	76 (47.2%)	49 (32.7%)	0.011
Smoking, *n* (%)				0.022
Never	172 (55.3%)	77 (47.8%)	95 (63.3%)	
Current	59 (19.0%)	35 (21.7%)	24 (16.0%)	
Prior	80 (25.7%)	49 (30.4%)	31 (20.7%)	
Other organ metastasis, *n* (%)	170 (54.7%)	91 (56.5%)	79 (52.7%)	0.569
Primary Organ, *n* (%)				0.099
Breast	37 (11.9%)	14 (8.7%)	23 (15.3%)	
Lung	71 (22.8%)	44 (27.3%)	27 (18.0%)	
Renal	40 (12.9%)	22 (13.7%)	18 (12.0%)	
Others	163 (52.4%)	81 (50.3%)	82 (54.7%)	
Time to last follow-up, mean ± SD	516.6 ± 634.1	489.0 ± 579.5	542.2 ± 682.9	0.568

Abbreviations: BMI: body mass index, HLD: hyperlipidemia.

**Table 2:** Preoperative variables and tumor characteristics.

	Total *N* = 311	ED *N* = 161	Clinic *N* = 150	*p*-Value
Mechanical Pain, *n* (%)	168 (54.0%)	75 (46.6%)	68 (45.3%)	0.909
Biologic Pain, *n* (%)	154 (49.5%)	97 (60.2%)	57 (38.0%)	<0.001
Neurologic Pain, *n* (%)	129 (41.5%)	58 (36.0%)	71 (47.3%)	0.050
Sensory Deficit, *n* (%)	92 (29.6%)	54 (33.5%)	38 (25.3%)	0.108
Motor Deficit, *n* (%)	142 (45.7%)	80 (49.7%)	62 (41.3%)	0.139
Strength, *n* (%)				0.533
0	27 (8.7%)	16 (9.9%)	11 (7.3%)	
1	6 (1.9%)	4 (2.5%)	2 (1.3%)	
2	26 (8.4%)	17 (10.6%)	9 (6.0%)	
3	24 (7.7%)	11 (6.8%)	13 (8.7%)	
4	66 (21.2%)	34 (21.1%)	32 (21.3%)	
5	153 (49.2%)	74 (46.0%)	79 (52.7%)	
Preop KPS, mean ± SD	66.2 ± 16.7	62.7 ± 17.75	70.0 ± 14.6	<0.001
Tumor Locations, *n* (%)				0.351
Cervical	40 (12.9%)	19 (11.8%)	21 (14.0%)	
Cervico-Thoracic	11 (3.5%)	5 (3.1%)	6 (4.0%)	
Thoraco-lumbar	184 (59.2%)	103 (64.0%)	81 (54.0%)	
Lumbar	76 (24.4%)	34 (21.1%)	42 (28.0%)	
Bilsky score, *n* (%)				0.049
0–1	80 (25.7%)	34 (21.1%)	46 (30.7%)	
2–3	218 (70.1%)	122 (75.8%)	96 (64.0%)	
Tumor Size, mean ± SD	1.7 ± 1.4	1.6 ± 1.3	1.8 ± 1.4	0.348
Oligometastatic, *n* (%)				0.049
1	216 (69.5%)	102 (63.4%)	114 (76.0%)	
<5	71 (22.8%)	45 (28.0%)	26 (17.3%)	
5+	24 (7.7%)	14 (8.7%)	10 (6.7%)	
Preoperative Embolization, *n* (%)	23 (7.4%)	16 (9.9%)	7 (4.7%)	0.088
Preop Chemo, *n* (%)	132 (42.4%)	60 (37.3%)	72 (48.0%)	0.066
Postop Chemo, *n* (%)	118 (37.9%)	56 (34.8%)	62 (41.3%)	0.244
Preop RT, *n* (%)	35 (11.3%)	13 (8.1%)	22 (14.7%)	0.074
Postop RT, *n* (%)	127 (40.8%)	70 (43.5%)	57 (38.0%)	0.357

Abbreviations: KPS: Karnofsky Performance Scale, Chemo: Chemotherapy, RT: Radiation.

**Table 3:** Intraoperative variables.

	Total *N* = 311	ED *N* = 161	Clinic *N* = 150	*p*-Value
Instrumented, *n* (%)	10 (3.2%)	155 (96.3%)	145 (96.7%)	>0.999
Decompressed, *n* (%)	292 (93.9%)	153 (95.0%)	139 (92.7%)	0.334
Total Decompressed Levels, mean ± SD	2.6 ± 1.3	2.7 ± 1.3	2.4 ± 1.3	0.041
Total Instrumented Levels, mean ± SD	5.3 ± 2.3	5.5 ± 2.3	5.1 ± 2.4	0.227
Transpedicular decompression, *n* (%)	164 (52.7%)	88 (54.7%)	76 (50.7%)	0.497
Costotransversectomy, *n* (%)	42 (13.5%)	15 (9.3%)	27 (18.0%)	0.031
Corpectomy/vertebrectomy, *n* (%)	164 (52.7%)	88 (54.7%)	76 (50.7%)	0.497
Operative Time, mean ± SD	315.2 ± 125.3	308.9 ± 98.9	322.0 ± 148.5	0.367
EBL (mL), mean ± SD	830.9 ± 882.2	905.9 ± 959.0	751.0 ± 787.6	0.122
Intraoperative Monitoring, *n* (%)	240 (77.2%)	124 (77.0%)	116 (77.3%)	>0.999
Intraoperative Monitoring Change, *n* (%)	6 (1.9%)	3 (1.9%)	3 (2.0%)	>0.999

Abbreviations: EBL: Estimated blood loss.

**Table 4:** Postoperative variables.

	Total *N* = 311	ED *N* = 161	Clinic *N* = 150	*p*-Value
Any Complication, *n* (%)	91 (29.3%)	47 (29.2%)	44 (29.3%)	>0.999
New Neurologic Deficit	15 (4.8%)	11 (6.8%)	4 (2.7%)	0.113
Cardiac Arrest	5 (1.6%)	5 (3.1%)	0	0.061
Hematoma	2 (0.6%)	1 (0.6%)	1 (0.7%)	>0.999
DVT	12 (3.9%)	9 (5.6%)	3 (2.0%)	0.141
UTI	14 (4.5%)	8 (5.0%)	6 (4.0%)	0.788
PE	8 (2.6%)	4 (2.5%)	4 (2.7%)	>0.999
Pneumonia	17 (5.5%)	7 (4.3%)	10 (6.7%)	0.458
Reintubate	4 (1.3%)	0	4 (2.7%)	0.053
Sepsis	14 (4.5%)	8 (5.0%)	6 (4.0%)	0.788
Stroke	3 (1.0%)	1 (0.6%)	2 (1.3%)	0.611
Mechanical Complication	9 (2.9%)	3 (1.9%)	6 (4.0%)	0.322
Wound complication	17 (5.5%)	7 (4.3%)	10 (6.7%)	0.457
Postop Radiculopathy	10 (3.2%)	6 (3.7%)	4 (2.7%)	0.751
LOS (Days), mean ± SD	6.9 ± 6.0	7.7 ± 6.1	6.1 ± 5.8	0.020
Postop disposition, *n* (%)				0.052
Floor	177 (56.9%)	83 (51.6%)	94 (62.7%)	
ICU	134 (43.1%)	78 (48.4%)	56 (37.3%)	
Discharge Home, *n* (%)	188 (60.5%)	84 (52.2%)	104 (69.3%)	0.025

Abbreviations: LOS: length of stay, DVT: deep vein thrombosis, UTI: urinary tract infection, PE: pulmonary embolism, ICU: intensive care unit.

**Table 5:** Short-term and long-term outcomes.

	Total *N* = 311	ED *N* = 161	Clinic *N* = 150	*p*-Value
Reoperation, *n* (%)	29 (9.3%)	13 (8.1%)	16 (10.7%)	0.443
Readmission, *n* (%)	70 (22.5%)	34 (21.1%)	36 (24.0%)	0.588
Local Recurrence, *n* (%)	33 (10.6%)	16 (9.9%)	17 (11.3%)	0.716
Time to local recurrence, mean ± SD	653.6 ± 839.2	870.0 ± 985.3	437.2 ± 641.6	0.424
KPS postop, mean ± SD	73.0 ± 16.8	72.4 ± 18.1	73.6 ± 15.4	0.608
KPS last follow-up, mean ± SD	64.7 ± 20.9	62.2 ± 21.5	67.1 ± 20.2	0.127
MMS postop, mean ± SD	1.7 ± 1.0	1.7 ± 1.0	1.6 ± 0.9	0.428
MMS last follow-up, mean ± SD	2.1 ± 1.1	2.2 ± 1.2	1.9 ± 1.1	0.160
Deceased, *n* (%)	202 (65.0%)	106 (65.8%)	96 (64.0%)	0.390
Time to death, mean ± SD	469.4 ± 676.1	458.2 ± 604.3	481.6 ± 749.9	0.005

Abbreviations: KPS: Karnofsky Performance Scale, MMS: modified McCormick Score.

**Table 6:** Linear/Logistic/Cox regression, controlling for age, comorbidities, preop KPS, primary organ of origin, and the presence of other organ metastasis.

		Univariate		Multivariate	
Perioperative Outcome	Independent Variable	β/OR/HR (95%CI)	*p*-Value	β/OR/HR (95%CI)	*p*-Value
LR	ED	0.86 (0.41–1.77)	0.690	1.18 (0.55–2.54)	0.665
Time to LR		0.68 (0.26–1.75)	0.426	0.98 (0.37–2.58)	0.980
Death		1.27 (0.78–2.06)	0.328	1.35 (0.81–2.25)	0.247
Time to Death		1.52 (1.13–2.06)	0.005	1.40 (1.01–1.93)	0.041
KPS Last		−4.90 (−11.23, 1.41)	0.127	−4.41 (−10.75, 1.91)	0.170

Abbreviations: LR: local recurrence, KPS: Karnofsky Performance Scale.

## Data Availability

Data for the results published in this paper are house in a secure network at Vanderbilt University Medical Center, and are available upon request.

## References

[1] Jacobs W.B., Perrin R.G. (2001). Evaluation and treatment of spinal metastases: An overview. Neurosurg. Focus.

[2] Miyazaki K., Kanda Y., Sakai Y., Yoshikawa R., Yurube T., Takeoka Y., Hara H., Akisue T., Kuroda R., Kakutani K. (2023). Effect of Bone Metastasis Cancer Board on Spinal Surgery Outcomes: A Retrospective Study. Medicina.

[3] Tarawneh A.M., Pasku D., Quraishi N.A. (2021). Surgical complications and re-operation rates in spinal metastases surgery: A systematic review. Eur. Spine J..

[4] Grant R., Papadopoulos S.M., Greenberg H.S. (1991). Metastatic epidural spinal cord compression. Neurol. Clin..

[5] Choi D., Bilsky M., Fehlings M., Fisher C., Gokaslan Z. (2017). Spine Oncology—Metastatic Spine Tumors. Neurosurgery.

[6] Patchell R.A., Tibbs P.A., Regine W.F., Payne R., Saris S., Kryscio R.J., Mohiuddin M., Young B. (2005). Direct decompressive surgical resection in the treatment of spinal cord compression caused by metastatic cancer: A randomised trial. Lancet.

[7] Zanaty A., George K.J. (2022). Outcomes and efficiency of managing patients admitted for surgery for spinal metastases. Surg. Neurol. Int..

[8] Chanbour H., Gangavarapu L.S., Chen J.W., Bendfeldt G.A., Younus I., Ahmed M., Roth S.G., Luo L.Y., Chotai S., Abtahi A.M. (2023). Unplanned Readmission After Surgery for Cervical Spine Metastases. World Neurosurg..

[9] Chen J.W., Chanbour H., Bendfeldt G.A., Gangavarapu L.S., Karlekar M.B., Abtahi A.M., Stephens B.F., Zuckerman S.L., Chotai S. (2023). Palliative Care Consultation Utilization Among Patient Undergoing Surgery for Metastatic Spinal Tumors. World Neurosurg..

[10] Chanbour H., Chen J.W.B., Gangavarapu L.S.B., Bendfeldt G.A.B., LaBarge M.E.B., Ahmed M., Roth S.G., Chotai S., Luo L.Y., Abtahi A.M. (2023). Unplanned Readmission Is Associated With Decreased Overall Survival and Performance After Metastatic Spine Surgery. Spine.

[11] Bendfeldt G.A., Chanbour H., Chen J.W., Gangavarapu L.S., LaBarge M.E., Ahmed M., Jonzzon S., Roth S.G., Chotai S., Luo L.Y. (2023). Does Low-Grade Versus High-Grade Bilsky Score Influence Local Recurrence and Overall Survival in Metastatic Spine Tumor Surgery?. Neurosurgery.

[12] Price M.J., De la Garza R., Dalton T., McCray E., Pennington Z., Erickson M., Walsh K.M., Yassari R., Sciubba D.M., Goodwin A.N. (2022). Insurance status as a mediator of clinical presentation, type of intervention, and short-term outcomes for patients with metastatic spine disease. Cancer Epidemiol..

[13] Davies J.M., Sleeman K.E., Leniz J., Wilson R., Higginson I.J., Verne J., Maddocks M., Murtagh F. (2019). Socioeconomic position and use of healthcare in the last year of life: A systematic review and meta-analysis. PLoS Med..

[14] Coussens L.M., Werb Z. (2002). Inflammation and cancer. Nature.

[15] Torisu H., Ono M., Kiryu H., Furue M., Ohmoto Y., Nakayama J., Nishioka Y., Sone S., Kuwano M. (2000). Macrophage infiltration correlates with tumor stage and angiogenesis in human malignant melanoma: Possible involvement of TNFalpha and IL-1alpha. Int. J. Cancer.

